# Measurement of Pulse Wave Signals and Blood Pressure by a Plastic Optical Fiber FBG Sensor

**DOI:** 10.3390/s19235088

**Published:** 2019-11-21

**Authors:** Yuki Haseda, Julien Bonefacino, Hwa-Yaw Tam, Shun Chino, Shouhei Koyama, Hiroaki Ishizawa

**Affiliations:** 1 Gratuate School of Medicine, Science and Technology, Shinshu University, 3-15-1 Tokida, Ueda, Nagano 386-8567, Japan; 19hs115f@shinshu-u.ac.jp; 2 Department of Electrical Engineering, Photonics Research Centre, The Hong Kong Polytechnic University, 11 Yuk Choi Road, Hung Hom, Kowloon, Hong Kong, China; jeebonef@polyu.edu.hk (J.B.); Hwa-yaw.tam@polyu.edu.hk (H.-Y.T.); 3 Interdisciplinary Graduate School of Science and Technology, Shinshu University, 3-15-1 Tokida, Ueda, Nagano 386-8567, Japan; 17st108j@shinshu-u.ac.jp; 4 Faculty of Textile Science and Technology, Shinshu University, 3-15-1 Tokida, Ueda, Nagano 386-8567, Japan; 5 Institute for Fiber Engineering, Shinshu University, 3-15-1 Tokida, Ueda, Nagano 386-8567, Japan; zawa@shinshu-u.ac.jp

**Keywords:** fiber Bragg grating, plastic optical fiber, non-invasive measurement, pulse wave signals, blood pressure, partial least squares regression

## Abstract

Fiber Bragg grating (FBG) sensors fabricated in silica optical fiber (Silica-FBG) have been used to measure the strain of human arteries as pulse wave signals. A variety of vital signs including blood pressure can be derived from these signals. However, silica optical fiber presents a safety risk because it is easily fractured. In this research, an FBG sensor fabricated in plastic optical fiber (POF-FBG) was employed to resolve this problem. Pulse wave signals were measured by POF-FBG and silica-FBG sensors for four subjects. After signal processing, a calibration curve was constructed by partial least squares regression, then blood pressure was calculated from the calibration curve. As a result, the POF-FBG sensor could measure the pulse wave signals with an signal to noise (SN) ratio at least eight times higher than the silica-FBG sensor. Further, the measured signals were substantially similar to those of an acceleration plethysmograph (APG). Blood pressure is measured with low error, but the POF-FBG APG correlation is distributed from 0.54 to 0.72, which is not as high as desired. Based on these results, pulse wave signals should be measured under a wide range of reference blood pressures to confirm the reliability of blood pressure measurement uses POF-FBG sensors.

## 1. Introduction

In recent years, the increase of medical expenses and lack of medical workers have become a social problem due to the aging society [1,2]. Therefore, self-management of health conditions and prevention of sickness by measuring vital signs continuously in daily life has become more important. However, current commercial measurement systems are not suitable for continuous measurement because the majority of them are for stationary use and apply physical constraints. For example, in the blood pressure measurement, a cuff is usually attached on the upper arm of subject. When the measurement begins, the cuff is pressured by injected air and then the arm of subject is also pressured. Thus, the subject feels pain and cannot take any movements during the measurement. To resolve those problems, various wearable vital sign measurement systems have been used globally [3,4,5,6,7]. For example, it was demonstrated that heart rate could be measured by photoplethysmography (PPG) using the Apple Watch^TM^ [3]. Using the PPG method, vital signs such as heart rate or stress can easily be measured by attaching sensors to the fingertip [4]. In addition, only a light emitting diode (LED) as the light source, a photodetector, and microcontroller are required to implement a measurement device. Therefore, it is easy to miniaturize measurement devices. However, there is no report that blood pressure and blood glucose level can be measured using PPG. Moreover, in the situation such as perspiration on the skin surface, the light intensity detected by the photodetector will decrease and measurement becomes difficult.

To resolve those problems, we are developing a non-invasive, wearable, and minimally constraining multi vital sign measurement system using a fiber Bragg grating (FBG) sensor. An FBG sensor is a small and light weight strain sensor fabricated in optical fiber, which has high sensitivity. In previous research, it is demonstrated that vital signs can be determined using pulse wave signals measured by a silica-FBG sensor [8,9,10,11,12,13]. However, silica optical fiber is easily broken by bending or extension. In addition, silica optical fiber produces sharp edges when break, representing a danger for the user, and prohibiting washing of the fiber and measurements during exercise. Therefore, there is a crucial need for safe FBG sensors for vital sign measurements.

In this report, an FBG sensor fabricated in plastic optical fiber (POF-FBG) sensor was used to resolve those problems. The plastic optical fiber is more flexible, resulting in resistance to bending and extension breaks, and a sharp cross section is not formed when breaks do occur. Furthermore, the plastic optical fiber is biocompatible because it is made from organic compounds like polymethyl methacrylate (PMMA) [14]. These features make it suitable for vital sign measurement. In addition, Bonefacino et al. reported the first demonstration of heartbeats measurements at the brachial artery location using POF-FBG [14]. It is further reported that use of a POF-FBG sensor results in a 20-fold sensitivity improvement over silica-FBG sensors. However, no attempt to calculate blood pressure from wavelength shifts measured by the POF-FBG sensor is made. In this report, we describe the results of measurement of pulse wave signals and the calculation of blood pressure from these signals.

## 2. Principle of the FBG Sensor

**Figure 1:**
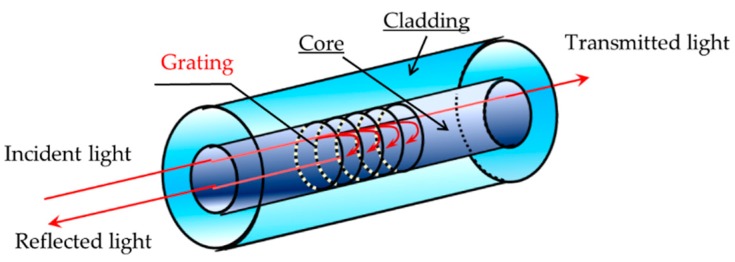
Schematic view of the sensor (fiber Bragg grating).

In this report, the FBG sensor system (SM130: Micron Optics, Inc., Atlanta, GA, USA) was used. This system is composed of a laser light source and an interrogator. The FBG sensor has a diffraction grating fabricated in the core of the optical fiber as shown in Figure 1. When broadband light is incident on the diffraction grating, only the specific wavelength corresponding to the Bragg wavelength is reflected, and the remaining light is transmitted. The Bragg wavelength is determined by the period of the diffraction grating and the effective refractive index as described by Equation (1).

(1) $$\lambda_{B}=2n_{eff}\Lambda$$

In Equation (1), *n_eff_* and Λ are the effective refraction index and the period of the diffraction grating, and *λ_B_* is the corresponding Bragg wavelength. When pressure is applied to the grating, the Bragg wavelength shifts because the period of the diffraction grating changes. The FBG sensor system measures pressure by detecting and calculating the shift of the Bragg wavelength.

**Figure 2:**
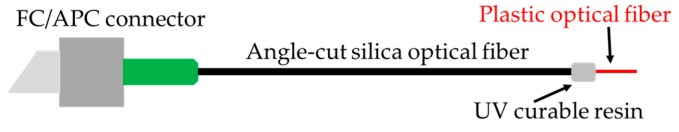
Schematic view of the optical fiber showing the fiber Bragg grating sensor fabricated in plastic optical fiber (POF-FBG) sensor.

Table 1 shows the core diameter, cladding diameter, and Bragg wavelength of the POF-FBG and silica-FBG sensors used in this report. As shown in Table 1, the Bragg wavelength of the POF-FBG sensor is in the near infrared. However, POFs have high attenuation in the IR region [14]. Therefore, a 50 mm POF was used, in which an FBG sensor was fabricated. The POF was attached to an angle-cut silica optical fiber as shown in Figure 2. One end of the silica fiber was cleaved with angle of ≈8° to avoid Fresnel reflection and glued to the POF using UV curable glue (NORLAND 78), ensuring low coupling losses and strong joint. The other end of the silica fiber was spliced to an ferule connector/angled physical contact (FC/APC) connector linked to the interrogator. Near infrared light incident on the silica optical fiber via the FC/APC connector can thus be propagated to the plastic optical fiber with low attenuation. As a result, the POF-FBG reflected Bragg wavelength has sufficient light power to be detected by the interrogator.

## 3. Experimental Methods

### 3.1. Measurement of Pusle Wave Signals and Reference Blood Pressure

**Figure 3:**
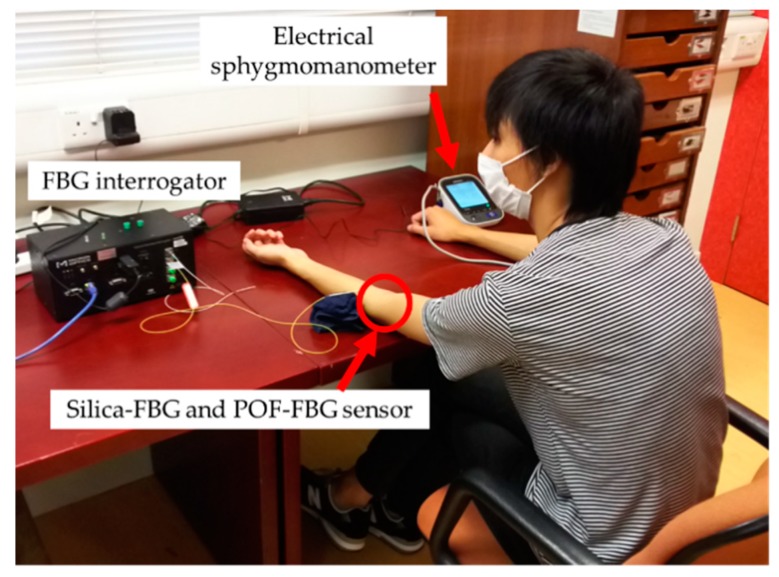
Experimental measurement of a subject.

Figure 3 shows a photograph of experimental measurement. In this report, both the pulse wave signals and reference blood pressure were measured simultaneously 120 times for each of four subjects. Subject A and subject B were healthy males in their 20s, and subject C and subject D were healthy males in their 30s and 40s, respectively. In previous research, it was shown that FBG sensors could measure pulse wave signals at various points of the human body because of its high sensitivity [8]. In addition, when the height of the blood pressure measuring point differs from that of the heart, the blood pressure was measured with a large error because of the effects of gravity. If the measuring point is higher than the heart, the blood pressure increases to supply the blood against gravity. Whereas, if the measuring point is lower than the heart, the blood pressure decreases because the supply of blood is assisted by gravity. To suppress these effects, the POF-FBG sensor was installed on the brachial artery of the left elbow in the seated position. In addition, the silica-FBG sensor was installed in close proximity to the POF-FBG sensor for comparison of the respective SN ratios and waveforms. Both the POF-FBG and silica-FBG sensors were attached to the brachial artery by surgical tape. Pulse wave signals were measured at a sampling frequency of 1 kHz, at which it was previously demonstrated that blood pressure could be measured by an FBG sensor [15].

The FBG sensor cannot measure the blood pressure directly. Therefore, the blood pressure was calculated using a calibration curve constructed for each pulse wave signal from the signal and a reference blood pressure [15]. Both systolic blood pressure (SBP) and diastolic blood pressure (DBP) were measured 120 times as the reference blood pressure at the right upper arm by an electrical sphygmomanometer (HEM-7510C: OMRON Corporation, Kyoto, Japan), which can measure the blood pressure with an accuracy of ±3 mmHg.

### 3.2. Processing and Analysis

In the processing and analysis of pulse wave signals, sixth processing was used for blood pressure prediction. These processing are (1) filtering processing, (2) first differentiation, (3) separation from peak to peak, (4) averaging, (5) normalization, and (6) blood pressure calculation by partial least squares regression (PLSR). These processing were performed from (1) to (6) in order.

Raw pulse wave signals include high frequency noise, which decreases the calculation precision of the blood pressure. Therefore, as in previous research, a band pass filter with a pass band from 0.5 to 5 Hz was used to reduce high frequency noise prior to blood pressure calculation [8,9,10,11,12,13].

**Figure 4:**
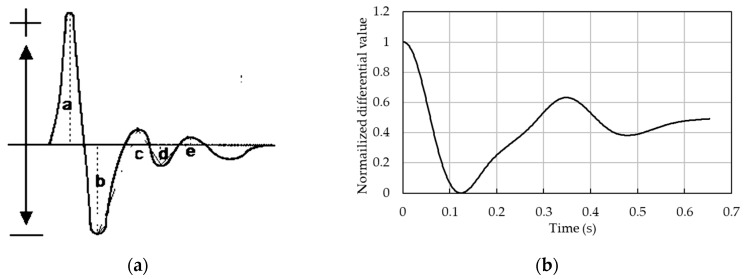
Comparison of acceleration plethysmograph (APG) and silica-FBG waveforms: (**a**) APG waveform and (**b**) silica-FBG sensor waveform.

Figure 4 shows the waveform of the acceleration plethysmograph (APG) and the waveform obtained by differentiation after the filtering process of previous research. APG is obtained by differentiating the volume plethysmograph, which shows volume fluctuations of the blood vessel. In addition, it is demonstrated that FBG sensors can measure pulse wave signals, which are similar to those of volume plethysmographs [15]. Extrema A–E are observed in the APG waveform. It is known that the heights of the extrema change when the blood pressure changes. Therefore, it is expected that the blood pressure can be calculated from the changes in height of the extrema. Thus, the first derivative was taken post-filtering.

After the first differentiation process, pulse wave signals were separated to acquire a single pulse wave corresponding to one heartbeat. Single pulse wave signals were acquired by cutting pulse wave signals from peak to the next peak. Next, the averaged pulse wave signals were acquired by averaging assembled single pulse wave signals. Then, normalized pulse wave signals were acquired by normalizing the averaged pulse wave signals along the horizontal axis. Each maximum and minimum value of the vertical axis was normalized to 1 and 0, respectively. The number of sampling points on the horizontal axis was unified in the fewest number of sampling points.

After normalization process, partial least squares regression (PLSR) was used to construct a calibration curve and predict the blood pressure from the processed FBG data. PLSR is a multivariate analysis method, in which the objective variables are regarded as having error. In addition, the reference blood pressure also has error. Furthermore, PLSR can be used to construct a reasonable calibration curve with fewer factors than principal components regression (PCR). Therefore, PLSR is suitable. Firstly, principal components analysis was performed on the pulse wave signals, and the feature vector called the PLS factor was calculated. Objective variables were represented by the linear combination of PLS factors acquired from explanatory variables. The optimal number of PLS factors were statistically tested at the 5% significance level. Finally, the calibration curve represented by the optimal PLS factors was used for calculation of the blood pressure. In this report, the explanatory variable was the normalized pulse wave signal, and the objective variable was the reference blood pressure. Four PLS factors were used to represent the objective variables. In this research, 120 data sets were prepared for PLSR by using raw data measured 120 times, which were composed of the normalized pulse wave signals and reference blood pressure. Eighty data sets were selected randomly and used for the construction of the calibration curve, and the remaining data sets were used for validation. For the evaluation of the error in the calibration curve and blood pressure calculation, the standard error of calibration (SEC) and the standard error of prediction (SEP) were used. SEC can be obtained by calculating the standard deviation of the difference between the reference blood pressure and calculated blood pressure by PLSR. In this calculation method, by using not the calculated blood pressure by PLSR but the calculated blood pressure by the calibration curve, SEP also can be obtained.

## 4. Experimental Results and Discussion

### 4.1. Pulse Wave Signals Measured by POF-FBG

**Figure 5:**
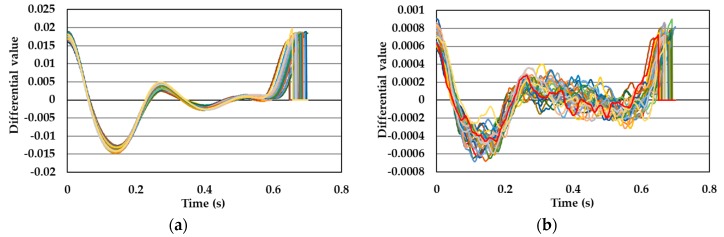
The single pulse wave signals of subject A: (**a**) POF-FBG sensor and (**b**) silica-FBG sensor.

**Figure 6:**
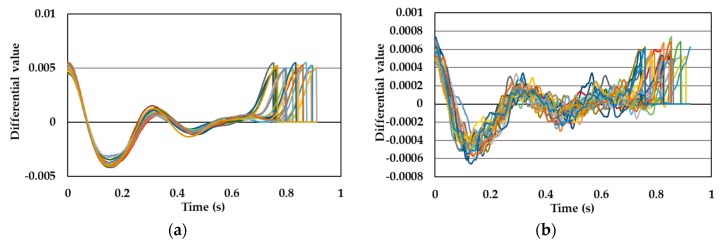
The single pulse wave signals of subject B: (**a**) POF-FBG sensor and (**b**) silica-FBG sensor.

**Figure 7:**
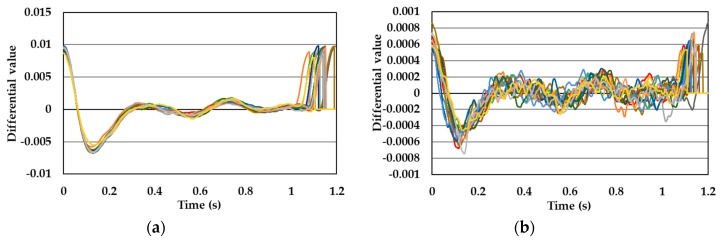
The single pulse wave signals of subject C: (**a**) POF-FBG sensor and (**b**) silica-FBG sensor.

**Figure 8:**
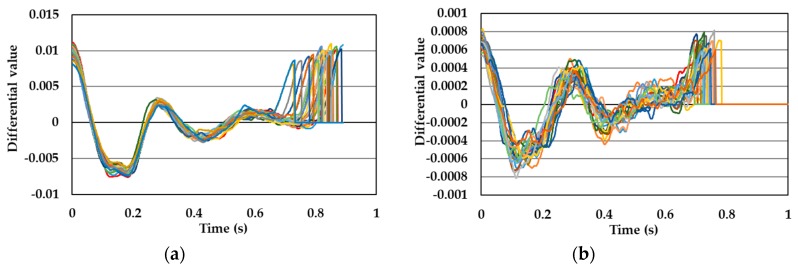
The single pulse wave signals of subject D: (**a**) POF-FBG sensor and (**b**) silica-FBG sensor.

Figure 5, Figure 6, Figure 7 and Figure 8 show single pulse wave signals measured by both the POF-FBG and Silica-FBG sensors in one measurement time for each of the four subjects, respectively. These single pulse wave signals were obtained after filtering, first differentiation, averaging, and the normalization process. As shown in the pulse wave signals of the silica-FBG sensor, the detailed waveform could not be determined because of high frequency noise remnants. However, the detailed waveform could be confirmed without the effect of noise in the pulse wave signals of the POF-FBG sensor. Therefore, it was demonstrated that the POF-FBG sensor could measure the pulse wave signals with a higher SN ratio compared to the silica-FBG sensor. In addition, the single pulse wave signals measured by the POF-FBG sensor were similar to the waveform of the APG shown in Figure 4. Thus, it was demonstrated that the POF-FBG sensor could also measure a waveform similar to the APG for an adequate SN ratio.

In this research, both the POF-FBG and silica-FBG sensors were attached to the same measuring points using surgical tape. Therefore, as shown in previous research, the SN ratio of the pulse wave signals were improved by at least eight times, due to the difference in sensitivity between the POF-FBG and silica-FBG sensors [14].

### 4.2. Calculation of Blood Pressure using Pulse Wave Signals

**Figure 9:**
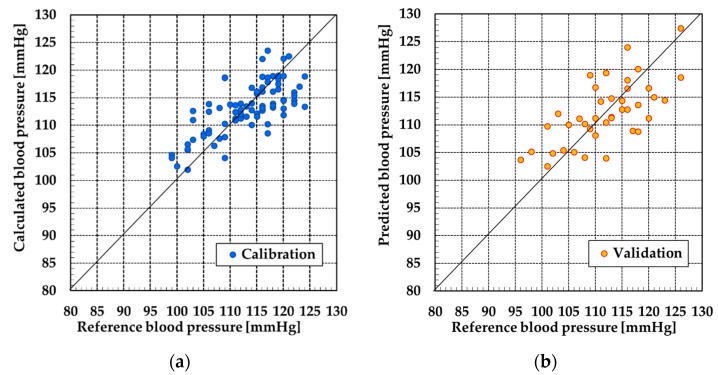
Result at systolic blood pressure (SBP) by partial least squares regression (PLSR) of subject A: (**a**) calibration and (**b**) validation.

**Figure 10:**
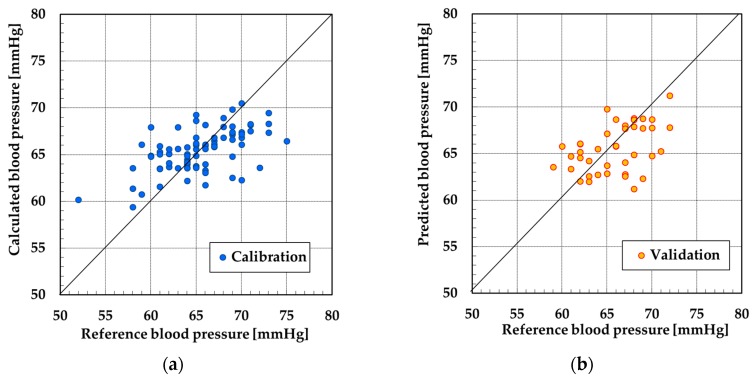
Result at diastolic blood pressure (DBP) by PLSR of subject A: (**a**) calibration and (**b**) validation.

**Figure 11:**
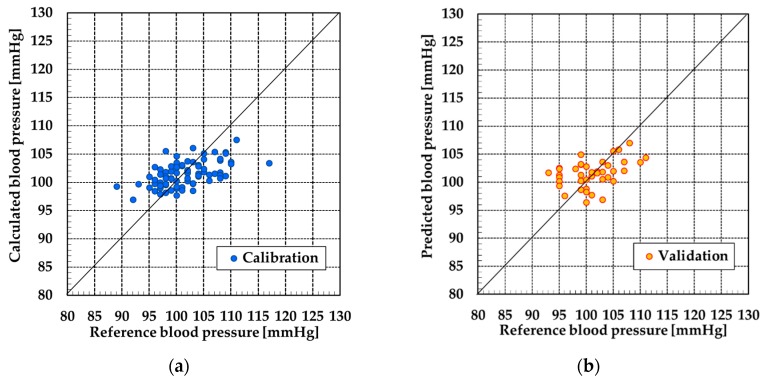
Result at SBP by PLSR of subject B: (**a**) calibration and (**b**) validation.

**Figure 12:**
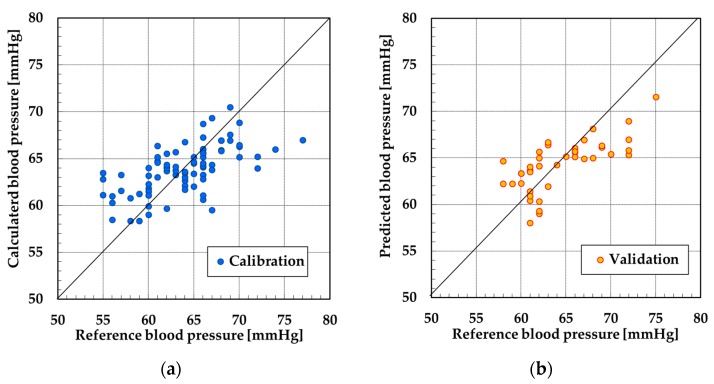
Result at DBP by PLSR for subject B: (**a**) calibration and (**b**) validation.

The data from subjects C and D could not be used because the SN ratio of pulse wave signals were poor. Therefore, the data from subjects A and B was used for PLSR. Figure 9, Figure 10, Figure 11 and Figure 12 show the results of calibration and validation of SBP and DBP. Table 2 shows the detail information of reference blood pressure in calibration and validation. Table 3 shows the correlation coefficient (R), SEC and SEP. In the calibration graph, the vertical axis indicates the calculated blood pressure using PLSR. In the validation graph, vertical axis indicates the blood pressure predicted by the calibration curve. Horizontal axis indicates the reference blood pressure in both calibration and validation graph.

For subject A, the correlation coefficient of the calibration curve exceeded 0.7 at SBP. However, the error exceeded 6 mmHg in validation. The SEC and SEP at DBP were smaller than the values at SBP. However, the correlation coefficient was only 0.58. For subject B, the SEC and SEP of both SBP and DBP were almost equal to the error of the electrical sphygmomanometer. However, similarly to the DBP of subject A, the correlation coefficient was not high for SBP or DBP.

### 4.3. Adequancy of Blood Pressure Measurement Using a POF-FBG Sensor

Judging from Section 4.1 and Section 4.2, pulse wave signals were measured at an adequate SN ratio by the POF-FBG sensor. However, although the SEC and SEP were adequately small, the correlation coefficient was poor, with the exception of subject A. For subject A, high correlation was confirmed between the reference blood pressure and blood pressure calculated by the calibration curve at SBP. To discuss these results, we focused on the range of the reference blood pressure and single pulse wave signals.

For subject A at SBP, the calibration and validation plots of Figure 9 were evenly distributed over the range of 25 mmHg. However, in the other calibration and validation results, almost all the plots were distributed over the range of 10 or 15 mmHg. When the range of the reference blood pressure was narrow, the validation of correlation between the reference blood pressure and calculated blood pressure over a wide range was difficult. Therefore, as a future task, it is necessary that the reference blood pressure be measured over a wide range to improve the correlation with the measured blood pressure.

**Figure 13:**
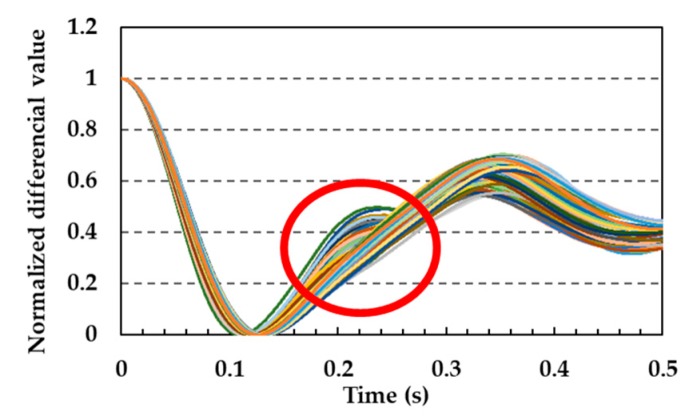
Reflected pulse wave signals (enclosed by circle).

**Figure 14:**
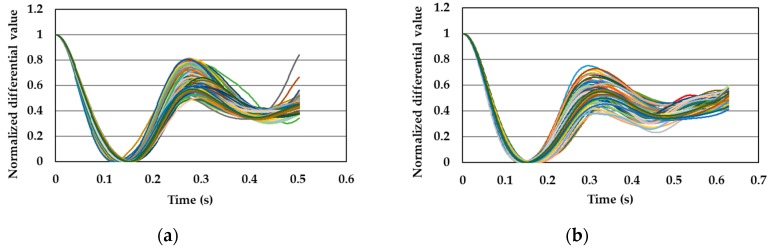
Normalized pulse wave signals: (**a**) subject A and (**b**) subject B.

Figure 13 shows all of the normalized pulse wave signals measured with the silica-FBG sensor for one subject from previous research. In this graph, a peak (reflected pulse wave signal) could be observed at approximately 0.22 s as indicated by the circle. Reflected pulse wave signals occur at points where the blood vessel divides into another vessel, or peripheral blood vessels exist. When the blood flow reaches these points, a component of the blood flow is reflected and is propagated to the measuring point, and the strain of blood vessel caused by the reflected pulse wave is detected by the FBG sensor. In addition, it is demonstrated that the reflected pulse wave signal significantly contributes to the calculation of the blood pressure in PLSR [16]. Figure 14 shows all of the normalized pulse wave signals for subjects A and B. As can be seen from these figures, the presence of reflected pulse wave signals between the first minima and second peak was not obvious. Reflected pulse wave signals had the characteristic that they were easily measured at points where peripheral vessels exist. Therefore, as a future task, pulse wave signals should be measured with reflected pulse wave signals so as to improve the accuracy of the calibration curve and calculation of the blood pressure.

## 5. Conclusions

In this paper, we focused on the validity of pulse wave signal measurement and calculation of blood pressure using a POF-FBG sensor. The pulse wave signals were measured by a POF-FBG sensor, and SBP and DBP were calculated by PLSR. In the measurement of pulse wave signals, the POF-FBG sensor was able to measure the pulse wave signals with SN ratio improved by at least eight times compared to the silica-FBG sensor for all subjects. Moreover, the single pulse wave signals measured by the POF-FBG sensor were significantly similar to the waveform of APG. Therefore, it was demonstrated that the POF-FBG sensor could measure pulse wave signals accurately and with very good repeatability.

On calculation of the blood pressure, a high correlation coefficient was confirmed at SBP for subject A. However, the correlation result at SBP for subject B was poor, as were the results at DBP for both subjects A and B. Regarding the accuracy of the calibration curve and the validation of blood pressure, the SEC and SEP at SBP of subject A were high. At SBP for subject B, and DBP for both subjects A and B, the SEC and SEP were almost equal to the accuracy of the electrical sphygmomanometer used in this report. To improve the correlation coefficient of the calibration curve, and accommodate a wide range of blood pressure measurements, it is necessary that the reference blood pressure be measured over a wide range in future experiments. In addition, the measurement of pulse wave signals that take reflected pulse wave signals into account should be performed to improve the accuracy of blood pressure measurement.

It is demonstrated that FBG sensor system can measure the pulse rate, respiratory rate, and blood pressure simultaneously and continuously [8,9,10,11,12,13]. By applying the flexible POF-FBG sensor to our FBG sensor system, we aimed for real-world implementation of safety FBG sensor system for users.

## Figures and Tables

**Table 1:** Fiber characteristics of silica-FBG and POF-FBG sensors.

	Core (µm)	Cladding (µm)	Bragg Wavelength (nm)
**Silica-FBG sensor**	8.2	125	1543
**POF-FBG sensor**	5.5	120	1553

**Table 2:** Details of reference blood pressure at calibration and validation.

Subject (Gender)	Cardiac Cycle	Number of Measurements	Value of Blood Pressure (mmHg)		
			Maximum	Minimum	Average
	**Calibration Data Sets**				
**A (male)**	SBP	80	124	99	113
	DBP	80	75	52	65
**B (male)**	SBP	80	117	89	102
	DBP	80	77	55	64
	**Validation Data Sets**				
**A (male)**	SBP	40	126	96	112
	DBP	40	72	59	66
**B (male)**	SBP	40	111	93	101
	DBP	40	75	58	64

**Table 3:** Calibration and validation results for each subject.

			Calibration		Validation
Subject	Cardiac Cycle	PLS Factor	R	SEC (mmHg)	SEP (mmHg)
**A**	SBP	4	0.72	5	6
	DBP	4	0.58	4	3
**B**	SBP	4	0.54	4	4
	DBP	4	0.63	4	3
